# Sub-Cutaneous Injection of Oxygen

**Published:** 1911-12

**Authors:** 


SUB-CUTANEOUS INJECTION OF OXYGEN. Felix Ramond, ibid., reports success with the method and cites Beraud's Thesis. A rubber balloon filled with oxygen is attached to a Pravaz syringe and the injection is made into the cellular tissues, as of the abdomen. The amount varies from a few c.c. for local asphyxia (gangrene) to 2 or 3 liters, repeated several times a day for general asphyxia. The author, on account of the difficulty of making even compression on the balloon, advises connecting it with a thermo-cautery bulb, the latter being refilled at its inlet from another balloon of oxygen.

